# Using bioinformatics for identifying and plugging metabolic pathway holes

**DOI:** 10.1073/pnas.2518071122

**Published:** 2025-09-02

**Authors:** Michael Y. Galperin

**Affiliations:** ^a^Computational Biology Branch, Division of Intramural Research, National Library of Medicine, National Institutes of Health, Bethesda, MD 20894

**Fig. 1.**
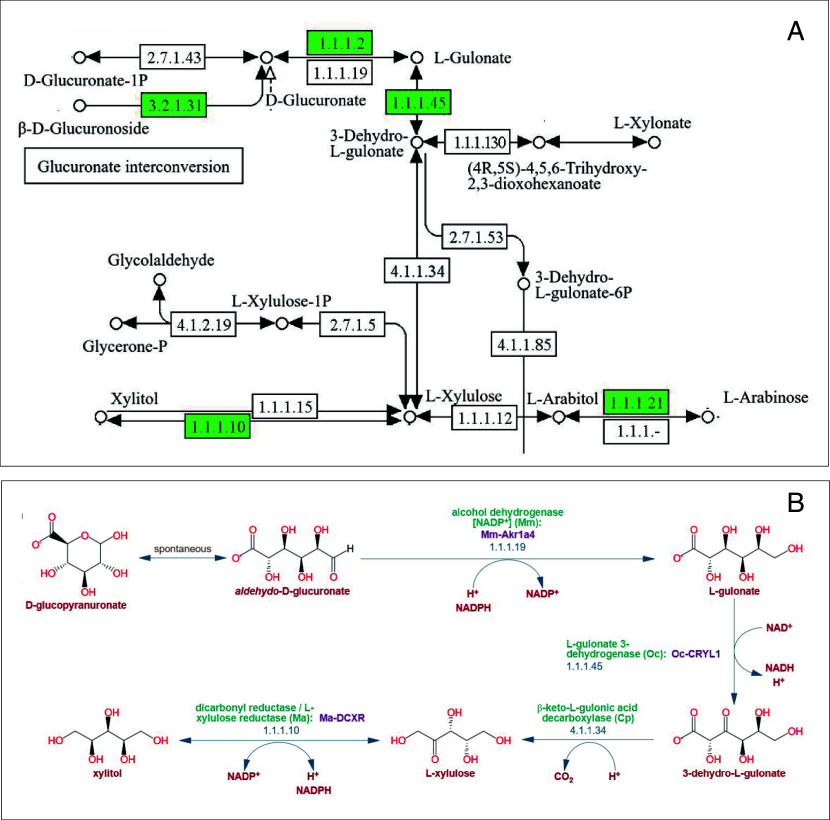
Pathway map representation for the conversion of D-glucuronate to xylitol. The maps were extracted from (*A*) KEGG: Kyoto Encyclopedia of Genes and Genomes (ref. 1) and (*B*) MetaCyc (ref. 2) databases. (*A*) KEGG pathway map “Pentose and glucuronate interconversions” (https://www.kegg.jp/pathway/map00040), mammalian version, with some compounds and reactions removed for clarity. The figures in boxes represent Enzyme Commission (EC) numbers; those assigned to mammalian genes are shaded green, missing and unassigned enzymes are shown on a white background. Conversion of L-gulonate to xylitol is catalyzed by L-gulonate 3-dehydrogenase (EC 1.1.1.45), producing 3-dehydro-L-gulonate (BKG); BKG decarboxylase (EC 4.1.1.34), producing L-xylulose; and L-xylulose reductase (EC 1.1.1.10), producing xylitol. Until the work of Malatesta et al. (3), EC 4.1.1.34 remained unidentified in the human genome. In the absence of L-xylulose reductase (EC: 1.1.1.10), L-xylulose cannot be metabolized and is excreted into urine, the condition referred to as pentosuria. (*B*) D-glucuronate degradation pathway map (https://metacyc.org/pathway?id=PWY-5525) in MetaCyc (2). The reactions catalyzed by EC 1.1.1.45 and EC 1.1.1.10 are annotated with their gene names, CRYL1 and DCXR, respectively. No gene is associated with EC 4.1.1.34 (*B*: Reprinted with permission from ref. 2).

The Biochemical Pathways chart, adorning professor offices all over the world, used to confuse and intimidate a lot of students. Why are there so many compounds with so many links between them? Am I supposed to learn all of them? How can anyone navigate this maze? In fact, this map, which until recently could be downloaded from the biochemical-pathways.com web site, is reportedly undergoing revision and is temporarily unavailable. Still, comprehensive pathway charts for many organisms are available online from such databases as KEGG (www.kegg.jp, ref. 1), Reactome (reactome.org), MetaCyc (metacyc.org, ref. 2), and others (Fig. 1). What many students might not realize is that not all biochemical reactions taking place in live organisms—including humans—are known; some reactions in those pathways are based on old experimental observations and have not been supported by any genetic (or genomic) data. This is exactly what happened in the case of 3-dehydro-L-gulonate (BKG) decarboxylase, an enzyme of arabinose and glucuronate metabolism, that was addressed in a paper by Malatesta et al. from the University of Parma, Italy, featured in this issue of PNAS (3).

While the overall pathway chart—and its fragments in biochemistry textbooks—may look as settled science, attempts to reconstruct the metabolic pathways based on genome sequence data encountered numerous problems, particularly in poorly studied bacteria, archaea, and single-celled eukaryotes (4–6). For many gene products, only a generic metabolic function, if any, could be predicted, and it was often not clear how the respective enzymes fit into the cellular machinery. Conversely, certain enzymatic reactions could not be associated with any specific gene; such reactions, commonly referred to as “orphan enzymes” or “pathway holes,” can be seen, among others, in pathways charts in the above-mentioned databases. There are even cases where an enzyme has been isolated (usually in the 1960s or 70s), biochemically characterized, and assigned an EC number, but has never been associated with any sequence (7). A recently updated version of the ORENZA database (8) at the University of Paris-Saclay in France (https://bioi2.i2bc.paris-saclay.fr/orenza/) specifically lists such orphan enzyme activities that do not have an associated amino acid sequence.

Obviously, there cannot be any real pathway holes: accumulation of almost any metabolite which is not converted into a limited number of amino acids, sugars, nucleotides, lipids, and other standard compounds that serve as building blocks for cellular components would upset cellular homeostasis and interfere with the cell growth; such metabolites must be promptly hydrolyzed or excreted from the cell. That is also a likely explanation for the complexity of metabolic pathways: all metabolic interconversions must occur without generating any toxic intermediates; a live cell that produces such intermediates would not be alive for too long and would be counterselected in the course of evolution. Indeed, genomic analyses identified a wide variety of enzymes that participate in detoxification of various side products of normal cell metabolism. These processes have been referred to as error prevention, error avoidance, house cleaning (9), or, more recently, metabolite damage prevention and repair (10).

Given these constraints, missing enzymes represent attractive targets for applying the whole arsenal of bioinformatics tools. Such enzymes could, in principle, comprise distant homologs of the enzymes catalyzing chemically similar reactions and could be identified through sequence similarity searches, as well as structural predictions and searches for related structures. Non-homology-based methods, making use of the vast genomic data, include, among others, analyses of 1) genomic neighborhoods in various organisms (operon organization of the gene’s orthologs in diverse bacteria), 2) domain fusions, 3) gene co-occurrence in multiple genomes, 4) gene coexpression, and 5) protein–protein interactions. However, in most cases, such analyses are being performed on a case-by-case basis to satisfy the specific needs of the project.

In this work, the Italian researchers chose to identify the pathway holes and suggest likely candidates for plugging those holes on a whole-genome basis. To do so, they constructed a sophisticated bioinformatics pipeline that relied on coevolutionary analysis introduced in their previous PNAS paper (11). This analysis is based on the well-known observations that functionally related genes typically show patterns of coevolution and coexpression (and sometimes colocalization). Thus, enzymes involved in the same pathway often show similar patterns of gene gain and loss across species (4). Accordingly, Malatesta et al. extracted from the OrthoDB database (12) families of orthologous proteins that had human representatives and calculated coevolution scores between various human metabolic enzymes. Applying these scores to the KEGG pathway charts, they were able to pinpoint those reactions within the same module that showed reliable connections to other human reactions but lacked associated sequence records in the KEGG Orthology database. From these reactions, the authors selected those that were sandwiched between two reactions already known to occur in humans and therefore represented likely pathway holes.

As a result of their analyses, Malatesta et al. came up with a list of 10 biochemical reactions that were known to take place in human cells but had no genes assigned to them. Three of these reactions (quinolinate hydrolysis in the NAD biosynthesis pathway, hydrolysis of pyrroline-5-carboxylate in the proline degradation pathway, and hydrolysis of phosphocreatine) could have occurred spontaneously and not necessarily needed a dedicated enzyme, although the authors suggested possible candidates to catalyze these reactions. Of the other six enzymes, representing genuine pathway holes, four had an assigned EC number, meaning that, at some point, the respective enzyme had been experimentally characterized.

To verify the validity of their bioinformatics pipeline, the authors tested one of these predictions, identification of 3-dehydro-L-gulonate (BKG) decarboxylase, a missing enzyme of L-arabinose and D-glucuronate metabolism, which converts the six-carbon BKG into five-carbon L-xylulose (Fig. 1). This enzyme was initially identified in 1961 (13) in the laboratory of Gilbert Ashwell at the NIH (14). Later that year, BKG decarboxylase was partially purified from guinea pig liver (15), assigned the EC number 4.1.1.34, but never characterized any further. In humans, L-xylulose gets reduced to xylitol, which is further metabolized into D-xylulose, then D-ribulose-5-phosphate, which enters the pentose phosphate pathway. In the absence of L-xylulose reductase (EC: 1.1.1.10), encoded by the dicarbonyl and L-xylulose reductase (DCXR) gene, L-xylulose gets released to the urine. This condition, seen primarily in Ashkenazi Jews, is referred to as pentosuria; it was initially confused with diabetes but is now considered benign.

BKG decarboxylase, and the gene encoding it, remained without further characterization for the past 65 years until this work identified it as C11orf54 (also known as PTD012; UniProt entry Q9H0W9; GenBank entry CAB66540), which is well conserved in eukaryotes, from mammals to fungi, and is also found in some bacteria. It belongs to the Domain of Unidentified Function family DUF1907 (PF08925) in the Pfam database and IPR015021 in InterPro (16, 17). Previous studies of this protein revealed its ability to hydrolyze *p*-nitrophenyl acetate (18), which led to the entire family being annotated as ester hydrolases. A high-resolution 3D structure of PTD012 (Protein DataBank entry 1XCR) shows an alpha–beta–beta–alpha four-layer topology with a bound Zn^2+^ ion, coordinated by three conserved His residues (18). This structure is related to the structures of bacterial acetolactate decarboxylases (e.g., PDB: 4BT4, RMSD of 3.0 Å over 210 aligned residues at 14% identity), which supported the assignment of BKG decarboxylase activity of PTD012, particularly given the similarity of respective substrates, BKG and acetolactate (3). Further support for the proposed BKG decarboxylase activity of PTD012 came from the analysis of domain fusions. While most vertebrate PTD012-like proteins represented stand-alone DUF1907 domains, three genera of rotifers, *Adineta*, *Didymodactylos*, and *Rotaria*, encoded fusions of DUF1907 with 3HCDH domains that code for various dehydrogenases, including L-gulonate 3-dehydrogenase (EC 1.1.1.45), the enzyme that catalyzes BKG formation, the previous step of the pathway (Fig. 1). These fusions supported a functional link between the two enzymes. This example illustrates the value of looking beyond just human and mammalian sequences: important clues can often be found in distantly related organisms. Finally, the involvement of C11orf54 in sugar metabolism was supported by the properties of its ortholog in *Drosophila melanogaster*, Meep, product of the *CG32335* gene, which had been implicated in insulin signaling (19). In flies reared on a high-sugar diet, knockdown of Meep caused hyperglycemia, reduced growth, developmental delay, pupal lethality, and reduced longevity (19). These observations convinced the authors that they were on the right track, and they performed the enzymatic measurements to verify the BKG decarboxylase activity of PTD012 (3).

Identification of C11orf54 as a beta-ketogulonate decarboxylase opens new avenues in studies of pentose metabolism and the potential moonlighting functions of this ORF.

Despite all this evidence, C11orf54 might have a different, possibly additional, activity when expressed in the cell nucleus. This protein has been implicated in regulation of cell proliferation and cisplatin-induced DNA damage and apoptosis and proposed as a marker of prostate and renal cancers (20, 21). This would suggest that, in addition to its role as a BKG decarboxylase, C11orf54 might have some moonlighting activity. This would not be totally surprising, as its pathway neighbor L-gulonate 3-dehydrogenase (EC 1.1.1.45) has a moonlighting function as rabbit eye λ-crystallin. Besides, read-through of the C11orf54 ORF can produce its fusions with the next gene, Med17 (CRSP6), coding for a subunit of the RNA polymerase II mediator complex; such fusions have been seen in humans (e.g., A0A1W2PRB8 in UniProt), macaque, and several other mammals.

Summing up, identification of C11orf54 as a BKG decarboxylase opens new avenues in studies of pentose metabolism and the potential moonlighting functions of this ORF. This result also validates the authors’ bioinformatics pipeline, which could be used to identify and eventually plug other pathway holes in human metabolism.
